# Three *Medicago MtFUL* genes have distinct and overlapping expression patterns during vegetative and reproductive development and *35S:MtFULb* accelerates flowering and causes a terminal flower phenotype in *Arabidopsis*

**DOI:** 10.3389/fgene.2015.00050

**Published:** 2015-02-19

**Authors:** Mauren Jaudal, Lulu Zhang, Chong Che, Joanna Putterill

**Affiliations:** The Flowering Lab, School of Biological Sciences, University of AucklandAuckland, New Zealand

**Keywords:** FRUITFULL, MtFUL, MtFTa1, FT, *Medicago*, *Arabidopsis*, flowering time, terminal flower

## Abstract

The timing of the transition to flowering is carefully controlled by plants in order to optimize sexual reproduction and the ensuing production of seeds, grains, and fruits. The genetic networks that regulate floral induction are best characterized in the temperate eudicot *Arabidopsi*s in which the florigen gene *FT* plays a major role in promoting the transition to flowering. Legumes are an important plant group, but less is known about the regulation of their flowering time. In the model legume *Medicago truncatula* (*Medicago*), a temperate annual plant like *Arabidopsis*, flowering is induced by prolonged cold (vernalization) followed by long day lengths (LD). Recent molecular-genetic experiments have revealed that a *FT-like* gene, *MtFTa1*, is a central regulator of flowering time in *Medicago*. Here, we characterize the three *Medicago* FRUITFULL (FUL) MADS transcription factors, MtFULa, MtFULb, and MtFULc using phylogenetic analyses, gene expression profiling through developmental time courses, and functional analyses in transgenic plants. *MtFULa* and *MtFULb* have similarity in sequence and expression profiles under inductive environmental conditions during both vegetative and reproductive development while *MtFULc* is only up regulated in the apex after flowering in LD conditions. Sustained up regulation of *MtFULs* requires functional *MtFTa1* but their transcript levels are not affected during cold treatment. Overexpression of *MtFULa* and *MtFULb* promotes flowering in transgenic *Arabidopsis* plants with an additional terminal flower phenotype on some *35S:MtFULb* plants. An increase in transcript levels of the *MtFULs* was also observed in *Medicago* plants overexpressing *MtFTa1*. Our results suggest that the *MtFULs* are targets of *MtFTa1*. Overall, this work highlights the conserved functions of *FUL-like* genes in promoting flowering and other roles in plant development and thus contributes to our understanding of the genetic control of the flowering process in *Medicago*.

## INTRODUCTION

Flowering time is an important adaptive trait in crop plants because of its major effect on plant yield and productivity (Jung and Muller, 2009; Putterill et al., 2013). However, the genetic network that regulates the transition to flowering is best understood in the small Brassicaceous annual weed, *Arabidopsis* (Srikanth and Schmid, 2011; Andres and Coupland, 2012). In *Arabidopsis*, at least six pathways transduce external and internal cues and regulate flowering time by converging on floral integrator genes such as *FT, SOC1,* and *LFY* (Srikanth and Schmid, 2011; Andres and Coupland, 2012). For example, extended winter cold (vernalization) leads to epigenetic silencing of an important floral repressor FLC (Kim et al., 2009). Thus, after winter, FLC inhibition of *FT* and *SOC1* is relieved, allowing the photoperiod pathway via the activator gene, *CO* to up-regulate them which promotes flowering in the long days (LDs) of spring. The commitment to flower is associated with the development of inflorescence meristem which grows indeterminately producing floral primordia on its flanks. Among the genes implicated to regulate reproductive meristem identity include *LFY*, *SOC1*, other MADS-box transcription factors *FUL, AGL24, AP1,* and *CAL* as well as the flowering repressor *TFL1* (Fornara et al., 2010; Srikanth and Schmid, 2011; Posé et al., 2012; Torti et al., 2012).

*Arabidopsis FUL* is a member of the euFUL clade, one of the three core eudicot clades, euFUL, euAP1 and AGL79, in the AP1/SQUA/FUL lineage of MADS-box genes (Litt and Irish, 2003; Berbel et al., 2012; Pabón-Mora et al., 2012). It has several functions through *Arabidopsis* development including flowering time control, inflorescence meristem identity and carpel development (Mandel and Yanofsky, 1995a; Hempel et al., 1997; Gu et al., 1998; Ferrándiz et al., 2000a; Melzer et al., 2008; Torti et al., 2012; Balanzà et al., 2014). The age and ambient temperature flowering-time pathways regulate *Arabidopsis FUL* expression via the action of SPL proteins and FT and LD photoperiods stimulate *FUL* expression (Wang et al., 2009; Kim et al., 2012; Balanzà et al., 2014). *FUL* has partially redundant roles with *SOC1* in promoting *Arabidopsis* flowering as single *ful* mutants have a mild delay to flowering, but the absence of *SOC1* function in the *ful soc1* double mutant causes a later flowering phenotype (Melzer et al., 2008; Torti et al., 2012; Balanzà et al., 2014). A recent report suggests that FUL might act by forming heterodimers with MADS proteins SVP and SOC1 to antagonize floral repression by FLC and SVP (Balanzà et al., 2014). The role of *FUL-like* genes in flowering-time control in other plants is not well understood overall, but progress is being made (Pabón-Mora et al., 2012). For example, the *AP1/FUL* gene from the basal eudicot opium poppy promotes the transition to flowering (Pabón-Mora et al., 2012) and *VRN1*, a monocot *AP1/FUL-like* gene, is an important regulator of flowering in response to vernalization in the temperate grasses wheat and barley (Trevaskis et al., 2007; Distelfeld et al., 2009).

Despite good progress, major gaps remain in our current understanding of flowering networks in many other plants such as the Fabaceae family (Putterill et al., 2013; Wong et al., 2014). Our study focuses on the small temperate forage legume model plant, *Medicago truncatula* (*Medicago*) as it offers a number of advantages including a sequenced genome and tagged mutant lines for forward and reverse genetic screens (Tadege et al., 2009; Young et al., 2011; Putterill et al., 2013). *Medicago* flowers much more rapidly in LD if these conditions are preceded by vernalization (Clarkson and Russell, 1975; Laurie et al., 2011). The results of our work and others indicate that a *Medicago FT* gene,* MtFTa1*, one of five *MtFT* genes, is expressed in response to vernalization and LD photoperiods and functions as a major integrator gene of both the vernalization and LD photoperiod pathways (Laurie et al., 2011; Yeoh et al., 2011, 2013; Jaudal et al., 2013). However, other key components such as the FLC/MAF clade of floral repressors are missing from *Medicago* and the photoperiod pathway appears to differ from the *Arabidopsis* model (Hecht et al., 2005; Putterill et al., 2013; Wong et al., 2014).

To identify additional genes in the *Medicago* flowering time network, we are taking forward and reverse genetic approaches (Jaudal et al., 2013, 2014; Putterill et al., 2013; Yeoh et al., 2013). In our recent reports, we identified three new early flowering mutants in *Medicago* (Jaudal et al., 2013; Yeoh et al., 2013). These *spring* mutants flower rapidly in LD conditions in the absence of vernalization. This correlates with the early onset of *FTa1* expression. The *spring* mutants all have retroelement insertions in or near *MtFTa1* which cause elevated expression of *MtFTa1* in LD conditions in the absence of vernalization.

Global microarray analysis of gene expression in leaves of the early flowering *Medicago spring1* mutant, indicated that a second gene with elevated transcript levels was the MADS-box gene *MtFULb*, one of three *MtFUL* genes named for their sequence similarity to* FUL* (Hecht et al., 2005; Yeoh et al., 2013). Increased *MtFULb* transcript levels were also observed in transgenic plants over expressing *MtFTa1* suggesting that *MtFTa1* might control the expression of *MtFULb* (Yeoh et al., 2013). This has resemblance with *Arabidopsis* where increased levels of *FUL* transcript were seen in leaves of plants over-expressing *FT* (Teper-Bamnolker and Samach, 2005). Consistent with being an FT target, *FUL* is required for very early flowering in *Arabidopsis* plants over expressing *FT* (Teper-Bamnolker and Samach, 2005).

While the functional role of *Medicago FUL* genes have not been reported, the pea *VEG1/PsFULc* ortholog of* MtFULc* has been well characterized (Berbel et al., 2012). *VEG1* groups in the AGL79 clade of the AP1/SQUA/FUL lineage (Berbel et al., 2012). *veg1* mutants have an extreme non-flowering phenotype. However, this is not due to a defect in floral induction or phase delay, but because *VEG1* is required for the specification of secondary inflorescence meristems that produce flowers (Berbel et al., 2012).

In this study, to investigate *MtFUL* function in flowering, we molecularly characterized the three *Medicago FUL-like* genes, *MtFULa*, *MtFULb* and *MtFULc,* and *MtFTa1* by gene expression profiling in wild type, *fta1* mutant and transgenic *35S:MtFTa1 Medicago* plants and by transformation into wild type *Arabidopsis*.

## MATERIALS AND METHODS

### BIOINFORMATICS

BLAST searches of *Arabidopsis* and *Medicago* protein databases were performed with *Arabidopsis* FUL (AtFUL) and three previously identified *Medicago truncatula* FUL-like sequences, MtFULa, MtFULb, and MtFULc (Hecht et al., 2005; Berbel et al., 2012). Alignment of the highest scoring sequences was performed using ClustalW in the Geneious software package [version 8.0.4 available from www.geneious.com (Biomatters, Ltd.)]. The phylogenetic tree was generated using full length protein sequences using the neighbor-joining (NJ) method via bootstrap resampling in the Geneious program. The accession numbers for the protein sequences used are as follows: *Medicago* MtFULa (Medtr2g461760), MtFULb (Medtr4g109830), MtFULc (Medtr7g016630), MtPIM (Medtr8g066260), MtBM5A (MTR_5g046790), ACJ84407; *Arabidopsis* AtFUL (At5g60910), AtAGL79 (At3g30260), and AtAP1 (At1g69120); *Pisum sativum* (garden pea) PsFULa (AAX69065), PsFULb (JN974186), and PsFULc/VEG1 (JN974184). These sequences were obtained from previous studies (Hecht et al., 2005; Berbel et al., 2012; Fourquin et al., 2013), JCVI *Medicago* genome assembly build Mt4.0^1^, TAIR^2^, and NCBI^3^.

### PLANT MATERIAL AND GROWTH CONDITIONS

*Medicago truncatula* (*Medicago*) wild type R108_C3 (R108), the *fta1 Tnt1* insertion mutant NF3307 and the *FTa1* over expression line *35S:FTa1* in the R108 background were used in this study (Laurie et al., 2011). R108 belongs to the *Medicago truncatula* Gaertn (barrel medic) ssp.* tricycla*. Scarification, germination, and seed vernalization (V) prior to growth in LD conditions (16 h of light/8 h of dark) and cultivation of *Medicago* plants were done as described previously (Laurie et al., 2011; Yeoh et al., 2013). For plants that were vernalized as seedlings and then grown in warm LD [depicted as vernalized seedlings in long days (VSLDs) conditions], seedling vernalization was done as described previously (Jaudal et al., 2014) with minor modifications such that the seedlings were grown in LD at 22°C for 11–14 days, then vernalized by exposure to cold at 4°C for 14 days in LD and then transferred to warm (22°C) LD conditions until they flowered. Flowering time of *Medicago* plants was measured in days after planting of germinated seeds unless otherwise indicated in the text.

*Arabidopsis thaliana* wild type Columbia (Col) and transgenic plants over expressing *MtFUL* (*35S:MtFULa, 35S:MtFULb,* and *35S:MtFULc*) in the Col background (this work) were used in this study. These overexpression gene constructs were made by amplifying the cDNAs of the *MtFUL* genes from R108 RNA and inserting these into the plant transformation vector, pB2GW7 (Karimi et al., 2002) to create expression clones using the GATEWAY TECHNOLOGY (GW) kit (Invitrogen, Corporation, USA) according to the manufacturer’s instructions. The forward and reverse primers, with the ATG translation start codon underlined, used for GW cloning are: GW_MtFULa GGGGACAAGTTTGTACAAAAAAGCAGGCTATATTATGGGGAGAGGAAGGGTG, GGGGACCACTTTGTACAAGAAAGCTGGGTAGGACTAATTAAGCATCCAAGGT; GW_MtFULb GGGGACAAGTTTGTACAAAAAAGCAGGCTAATAATGGGGAGGGGAAGAG, GGGGACCACTTTGTACAAGAAAGCTGGGTCAAATGTACGTAATTATCTTTTTCTC; GW_MtFULc GGGGACAAGTTTGTACAAAAAAGCAGGCTTCATTCATCATCATGGGAAGGG, GGGGACCACTTTGTACAAGAAAGCTGGGTATCTAGTTGGTGAGATGATGGAG. *Arabidopsis* Col plants were infiltrated with *Agrobacterium* GV3101 carrying the constructs. T1 and T2 seeds were sterilized for 5 min in 4% bleach, washed, and stratified at 4°C for 3 days prior to planting on rock wool or soil with nutrient media and grown in LD conditions. Transgenic plants were selected by spraying with Basta. Plants were genotyped by PCR to confirm the presence of the *35S:MtFULa*, *35S:MtFULb*, *35S:MtFULc,* and the *Basta* transgenes. Genotyping primers for *35S:MtFULa* transgenic plants were 35S-F CACTGACGTAAGGGATGACG with MtFula_TC182438-R TGGGCGTTGCCATGGGTTTGAC; for *35S:MtFULb* the GW_MtFULb primers above were used; for *35S:MtFULc* the primers were newqRT-F AGGGCAAGGACATTGCAGGAGCA and newqRT-R TGGTGGTAGCACCTCTGGCTGACAA, and for *Basta* the primers were Basta F-2 GCGTTCAAAAGTCGCCTAAG and Basta-R GAAGTCCAGCTGCCAGAAAC. Flowering time of *Arabidopsis* plants was measured in total number of rosette and cauline leaves at flowering.

### RNA EXTRACTION AND REVERSE TRANSCRIPTASE-qPCR (RT-qPCR) ANALYSIS

RNA extraction, cDNA synthesis, and RT-qPCR were performed on *Arabidopsis* and *Medicago* samples as previously described (Laurie et al., 2011; Yeoh et al., 2013). The identity of the PCR amplicons was checked by DNA sequencing. In the *Medicago* developmental time courses, flower buds were small unopened flowers, while flowers were open flowers. *Medicago* gene expression is presented as mean ± SE, where *n* = 3 biological replicates (unless stated otherwise). Each replicate consists of pooled material from 3 individual plants. For *MtFTa1* and *MtFULb* expression in leaf under VSLD, the time points of 45 and 62 days are derived from 1 biological replicate. The *Medicago* data were normalized to the housekeeping gene, *protodermal factor 2* (*PDF2*). The forward and reverse primers used are PDF2: GTGTTTTGCTTCCGCCGTT, CCAAATCTTGCTCCCTCATCTG; MtFTa1: GTAGCAGTAGGAATCCACTAGC, ACACTCACTCTCGGTTGATTTCC; MtFULa: GGCCCAACTTGAGCAGCAAAATGAGG, TGGGCGTTGCCATGGGTTTGAC; MtFULb: AGAGCACGCAAAACTCAAGGCT, AGCTCTTTGAGACCTAAACCATCCAA; MtFULc: AGGGCAAGGACATTGCAGGAGCA, TGGTGGTAGCACCTCTGGCTGACAA. The *Arabidopsis* data were normalized to the housekeeping gene At2g32170 amplified using the primers TCCTTTTTCATCGACACTGC and CCATATGTGTCCGCAAAATG. *Arabidopsis* gene expression is derived from one sample consisting of ∼3 cauline leaves from the same plant and presented as mean ± SE, where *n* = 3 PCR technical replicates.

## RESULTS

### ANALYSIS OF THE SEQUENCE OF THREE *MtFUL-LIKE* GENES IN *Medicago*

**FIGURE 1:**
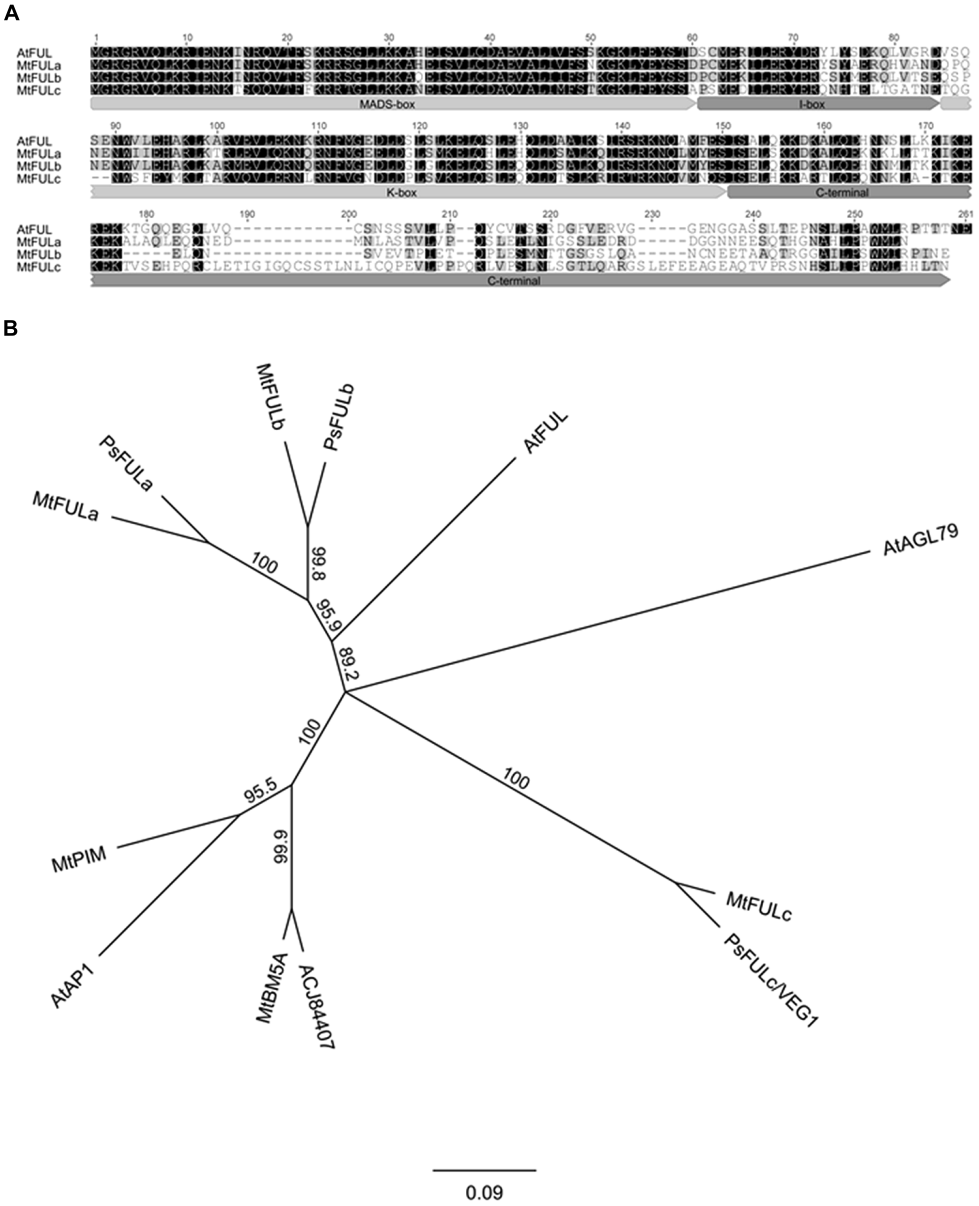
**Alignment and phylogenetic analysis of FUL - like proteins from *Medicago*, pea, and *Arabidopsis*. (A)** Alignment of the predicted MtFULa, MtFULb, MtFULc, and AtFUL protein sequences. Typical MIKC domains of MADS box transcription factors are marked. Amino acids with black shading are identical in all sequences, those in gray are similar residues **(B)** A consensus phylogenetic tree based on the full-length amino acid alignment of MtFULa, MtFULb, MtFULc, FUL - like proteins, and related MADS box transcription factors. The tree was generated using the neighbor-joining (NJ) method via bootstrap resampling with support threshold of 55%. The numbers indicate the bootstrap values based on 1000 replicates. At: *Arabidopsis thaliana*, Mt: *Medicago truncatula*, Ps: *Pisum sativum*.

Three *Medicago FUL-like* sequences (*MtFULa*, *MtFULb,* and *MtFULc*) were previously identified in Medicago EST and Genomic databases (Hecht et al., 2005). However, translation of the *MtFULb* sequence indicated that it encoded a protein of 148 amino acids (aa) that was truncated at the C-terminus compared to *Arabidopsis* FUL (FUL) and the other MtFULs. To investigate the transcript sequence further, we carried out 3′RACE. This indicated that there was an error in the Genbank sequence and that the corrected sequence encoded a protein of 232 aa which is comparable in size to FUL and MtFULa (242 aa and 236 aa, respectively) and MtFULc which is slightly longer at 256 aa. FUL shares 64.4% identity with MtFULa, 64.6% identity with MtFULb and 47.1% identity with MtFULc. MtFULa and MtFULb are 69.1% identical to each other and 48.2 and 49.4% identical to MtFULc, respectively. MtFULa, MtFULb, and MtFULc are predicted to encode MADS transcription factors as they have a conserved MADS-box domain, intervening (I-box) region, and keratin-like (K-box) motif (Parenicova et al., 2003; **Figure 1A**). The first few amino acids in the C-terminal region are quite conserved among *Arabidopsis* FUL and MtFUL proteins but the remainder of the sequences are divergent except for the L/MPPWML motif near the very end of the region, which was shown to be conserved in other eudicot FUL sequences (Litt and Irish, 2003).

Next, we performed reciprocal BLAST searches of *Arabidopsis* and *Medicago* protein databases with FUL and the three MtFUL-like protein sequences. The top scoring hits with *Arabidopsis* FUL in the *Medicago* databases were MtFULa, followed by MtFULb, MtPIM (AP1 ortholog; Benlloch et al., 2006), an additional MADS factor MtBM5A then MtFULc. MtFULa and MtFULb identified each other and AtFUL as top hits in BLAST searches of both databases. MtFULc identified AtFUL as top hit followed by MtBM5A, MtFULb, MtFULa, and MtPIM. This analysis overall confirmed that MtFULa and MtFULb are the most similar proteins to AtFUL in the *Medicago* databases.

A previous study conducted a phylogenetic analysis of genes that belong to the AP1/SQUA/FUL family, which included the MtFUL-like protein sequences (Berbel et al., 2012). Here, because we identified the full-length *MtFULb* sequence, in contrast to the truncated version in the GenBank database, and discovered two additional MADS-box genes (MtBM5A and ACJ84407) as a result of BLAST searches with FUL against the recently published *Medicago* sequence (Mt 4.0 version), we performed a simpler phylogenetic analysis incorporating these changes and including only the top scoring BLAST hits from *Arabidopsis* and *Medicago*, and some closely related pea sequences (**Figure 1B**). Our neighbor joining tree indicates that MtFULa and MtFULb form a sister clade to AtFUL, while MtFULc is more distantly related. All the three *Medicago* FULs have closely related pea sequence counterparts. MtBM5A and ACJ84407 form a sister clade to AP1 and MtPIM.

### GENE EXPRESSION PATTERNS OF *MtFUL* GENES IN A DEVELOPMENTAL TIME COURSE IN WILD TYPE *Medicago* IN LONG DAY CONDITIONS

**FIGURE 2:**
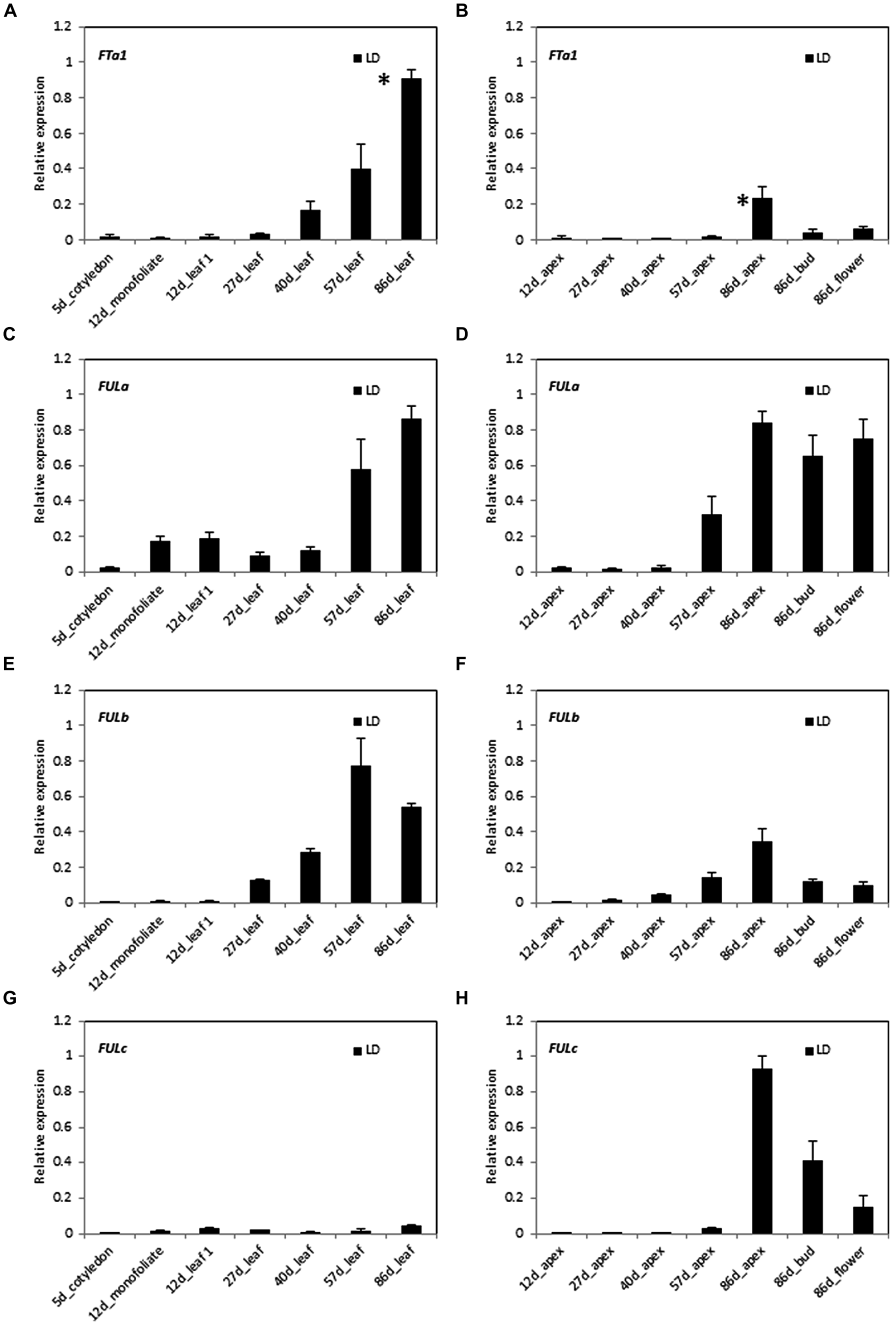
**Developmental regulation of *MtFTa1* and the three *MtFUL* genes in *Medicago* wild type R108 plants in long day (LD) conditions.** Relative gene expression levels in cotyledons, monofoliate leaves or trifoliate leaves **(A,C,E,G)** and uppermost apical buds, flower buds, or open flowers **(B,D,F,H)** at the times shown in days after planting germinated seeds in LD. The * shown in **(A,B)** indicate that plants had undergone the transition to flowering with floral buds first visible at 69 days after planting of germinated seeds. Tissues were harvested at ZT2. Gene expression was determined using RT-qPCR and the data are shown as the mean ± SE of three biological replicates, which were normalized to *PDF2*. The data is presented relative to the highest value in all tissues for each gene.

In order to investigate the potential roles of *MtFULa*, *MtFULb,* and *MtFULc* genes in *Medicago* flowering time regulation, we compared the expression of these genes and the floral integrator gene *MtFTa1* through a developmental time course in LD conditions (**Figure 2**). Gene expression profiles were determined by RT-qPCR on leaves, shoot apices, flower buds, and flowers. Flowering occurred at ∼69 days after planting germinated seeds in these conditions.

*MtFTa1* was slowly up regulated through development prior to flowering in these non-vernalized LD plants as reported previously (**Figures 2A,B**; Laurie et al., 2011). Its abundance began to noticeably increase in the 27 day-old trifoliate leaf and then rose at each time point thereafter with the highest level detected in the leaf of 86 day-old plants. Expression of *MtFTa1* in the shoot apex was much reduced compared to the leaf, with the highest level observed in the 86 day-old apex and reduced amounts in 86 day-old flower buds and flowers.

The *MtFULa* expression profile had some similarities with *MtFTa1* in that its transcript increased prior to flowering, particularly at 57 days with maximum levels detected in the 86 day-old leaf, apex, flower buds, and flowers (**Figures 2C,D**). However, it was detectable in leaves earlier than *MtFTa1*, including the monofoliate, which is the first true leaf. It was expressed at lower relative levels in shoot apices early on, but showed a strong increase in level at 57 days and continued to rise in the shoot apex, flower buds, and flowers of 86 day-old plants.

Of the three *MtFUL* genes, *MtFULb* had the most similar pattern of expression to *MtFTa1* in LD (**Figures 2E,F**). Its expression strongly increased at 27 days in the leaves and continued to rise to its peak at 57 days then slightly decreased in the 86 day-old plants. In the apical samples, transcript levels increased over time with the highest level detected in the 86 day-old shoot apices, but with lower levels detected in flower buds and flowers. Like *MtFTa1*, *MtFULb* transcript levels in apices were generally lower than in leaves at the same time points.

*MtFULc* was expressed in a strikingly different pattern to *MtFTa1*, *MtFULa,* and *MtFULb*. It was expressed at very low, but detectable levels in leaves throughout development (**Figures 2G,H**). It was first detectable in shoot apices at 57 days, but showed a very strong elevation in expression in shoot apices of 86 day-old flowering plants and was expressed at high, but slightly reduced relative levels in flower buds and open flowers.

Overall, in summary in LD conditions, transcripts of *MtFTa1*, *MtFULa,* and *MtFULb* were up regulated in leaves and shoot apices prior to the transition to flowering, while *MtFULc* transcript strongly increased in shoot apices after flowering.

### GENE EXPRESSION PATTERNS OF *MtFUL* GENES IN A DEVELOPMENTAL TIME COURSE IN WILD TYPE *Medicago* IN VERNALIZED LONG DAY CONDITIONS

**FIGURE 3:**
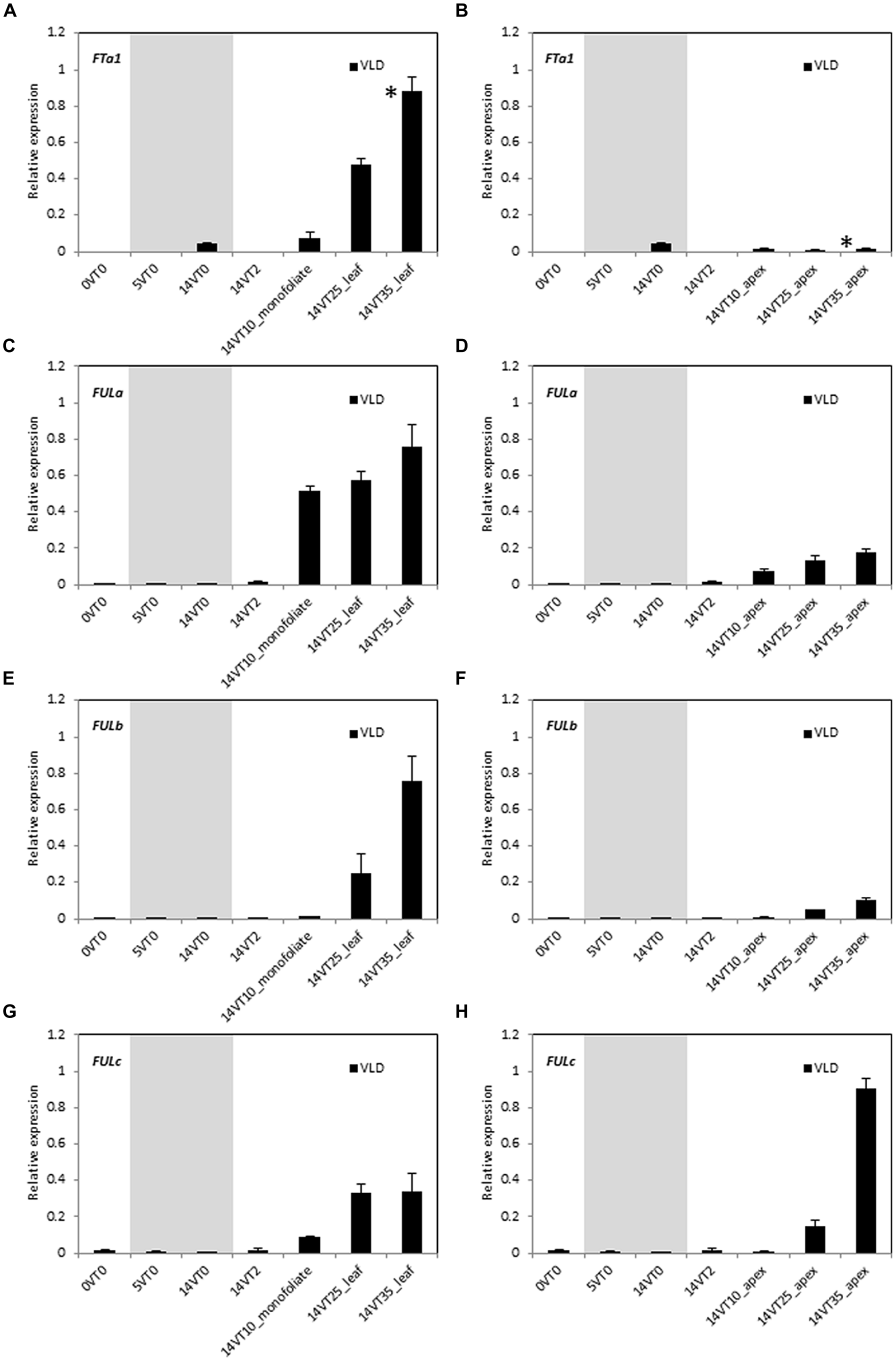
**Developmental regulation of the three *MtFUL* genes and the floral integrator gene *MtFTa1* in *Medicago* wild type R108 plants in LD conditions after vernalization of germinated seeds.** After germination (0VT0), *Medicago* seeds were vernalized by exposure to 2 weeks of cold at 4°C in the dark and then planted in pots and transferred to warm (22°C) LD conditions (VLD). Relative gene expression levels in whole seedlings, monofoliate leaves or trifoliate leaves **(A,C,E,G)** and uppermost apical buds **(B,D,F,H)** before, during and after vernalization are shown (e.g., 14VT2 signifies 14 days of vernalization and 2 days in warm LD conditions). Tissues were harvested from plants of increasing developmental ages grown in LD. The * shown in **(A,B)** indicate when plants had undergone the transition to flowering with floral buds first visible at 32 days after planting of the vernalized seeds. Tissues were harvested at ZT2. The gray shading indicates the 14 day vernalization treatment. Gene expression was determined using RT-qPCR and the data are shown as the mean ± SE of three biological replicates, which were normalized to *PDF2*. The data is presented relative to the highest value in all tissues for each gene.

Vernalization accelerates flowering in LD-grown *Medicago* wild type R108 plants (Laurie et al., 2011; Yeoh et al., 2013). To test if the expression of *MtFUL* genes were affected by vernalization, germinated seeds were exposed to the cold for 2 weeks followed by planting in soil and growth in warm LD conditions (VLD, **Figure 3**). This treatment promoted the transition to flowering with plants flowering at ∼32 days after planting. Transcripts of all of the four genes were barely detected in the newly germinated seeds or during the cold treatment, but expression levels began to rise after few days of growth in warm LD conditions prior to flowering.

As expected, *MtFTa1* transcript level showed a prominent increase in the 25 and 35 day-old leaves, but was barely detectable in the shoot apex (**Figures 3A,B**; Laurie et al., 2011). *MtFULa* transcript level showed a sharp rise, earlier than *MtFTa1* and *MtFULb,* and was greatly increased even in the monofoliate leaf after 10 days in LD conditions. Expression was not as strongly elevated in the apical samples as in the leaves, but a similar profile of expression was seen in the shoot apices (**Figures 3C,D**). The earlier onset of *MtFULa* expression in VLD compared with other genes was similar to the results for LD-grown plants (**Figure 2**). *MtFULb* transcript again showed a quite similar profile to *MtFTa1* showing a sharp rise in the 25 day-old leaf (**Figure 3E**). It was elevated in the apex at 25 and 35 days but at lower levels than in the leaf samples (**Figures 3E,F**). *MtFULc* transcript was most abundant in the 35 day-old apex after flowering, but was detected prior to the transition to flowering, in the 25 day-old apex (**Figures 3G,H**). The abundance in leaves relative to the apical samples increased in VLD compared to LD.

### GENE EXPRESSION PATTERNS OF MtFUL GENES IN A DEVELOPMENTAL TIME COURSE IN WILD TYPE AND fta1 MUTANT *Medicago* IN VERNALIZED SEEDLINGS IN LONG DAY CONDITIONS

**FIGURE 4:**
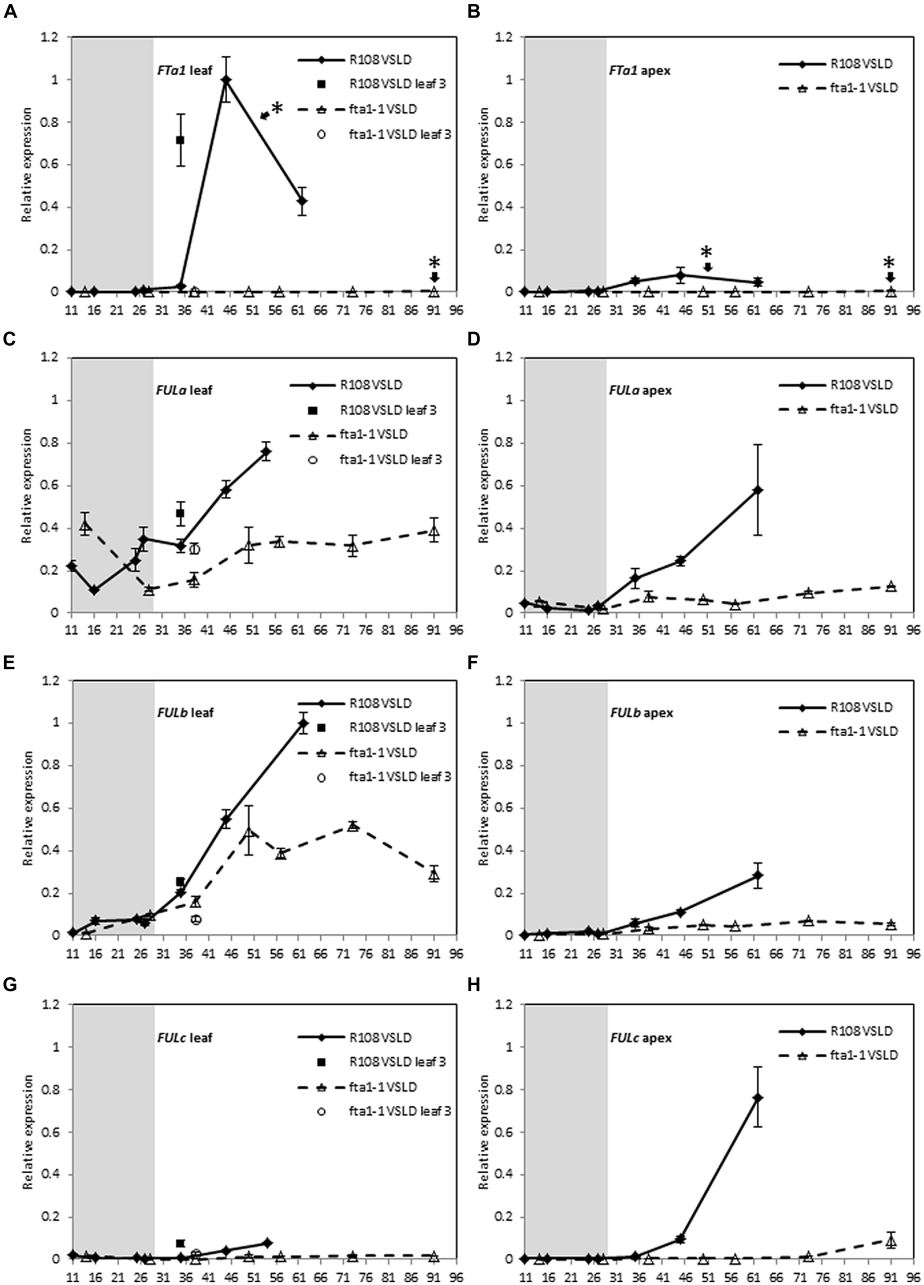
**Developmental regulation of the three *MtFUL* genes and the floral integrator gene *MtFTa1* in *Medicago* wild type R108 plants and the *fta1* mutant line in LD conditions after vernalization of young seedlings.** Seedlings were grown in LD for 11–14 days, then vernalized by exposure to cold for 14 days and then transferred to warm (22°C) LD conditions [vernalized seedlings in long days (VSLDs)]. Relative gene expression levels in trifoliate leaves **(A,C,E,G)** and uppermost apical buds **(B,D,F,H)** during the 14 day vernalization treatment (gray-shaded region) and after transfer to warm LD conditions are shown. Tissues were harvested from plants of increasing developmental ages (shown on the x-axis as days after planting of germinated seeds in the soil). The * shown in **(A,B)** indicate that floral buds were first visible on R108 plants at 52 days after planting of the germinated seeds while 91 days for the *fta1* mutant. Tissues were harvested at ZT2. Gene expression was determined using RT-qPCR and normalized to *PDF2*. The data are shown for R108 as the mean of one to three biological replicates relative to PDF2 with SE of the replicates or technical errors in cases of one biological sample. For *fta1* plants, gene expression is presented as the mean of three biological replicates relative to PDF2. Error bars are SE of the replicates. The data is presented relative to the highest value in all tissues for each gene.

To examine the dependence of *MtFUL* gene expression on *MtFTa1*, we compared the expression of *MtFULa, MtFULb,* and* MtFULc* in wild type and* fta1* mutant plants over a developmental time course (**Figure 4**). Seeds of both genotypes were germinated and grown in LD until they were 11–14 days old, then the seedlings were exposed to the cold for 2 weeks in LD and shifted to warm LD conditions (VSLD). Seedling vernalization provided us with the opportunity to test the effect of cold treatment directly on gene expression in young plants, rather than germinated seeds as in the previous experiment. In these conditions, the R108 wild type plants flowered at ∼52 days after the planting of germinated seeds, while the *fta1* mutants flowered much later when they were 91 days old. *MtFTa1* levels showed a dramatic increase in leaves of wild type plants after the plants were transferred to warm LD conditions and prior to flowering (**Figure 4A**). Expression levels were very low during the cold treatment of the young seedlings. A very modest increase in expression was detected in apices after plants were grown in warm LD conditions. No *MtFTa1* expression was detected in the *fta1* mutant. These results are consistent with previous work (Laurie et al., 2011).

Almost similar levels of* MtFULa* and *MtFULb* transcripts were observed in the leaves and apices of wild type and the *fta1* mutant early in development and during vernalization treatment, with no direct effect of cold on their transcript levels (**Figures 4C–F**). However, after subsequent growth in warm LD conditions, the expression patterns of *MtFULa* and *MtFULb* began to diverge between the wild type and *fta1* mutant. *MtFULa* and *MtFULb* were continuously up regulated in wild type, which correlated with the increase in *MtFTa1* accumulation in the leaves, while expression in the mutant plateaued off. Although the striking difference between *MtFTa1* levels in wild type and *fta1* mutant was not completely mirrored in the *MtFULa* and *MtFULb* profile in leaves, it is evident from the results that *MtFTa1* is needed for sustained upregulation of *MtFULa* and *MtFULb.* The maximum expression level of both genes was lower in the* fta1* mutant compared to wild type in the leaves, and this difference is more pronounced in the shoot apex. In contrast, *MtFULc* expression levels were very dependent on functional *MtFTa1* with almost no expression detected in the *fta1* mutant throughout development (**Figures 4G,H**). A very slight rise in transcript level was seen in the apical samples of *fta1* mutant at the time of flowering. There was no direct effect of cold on *MtFULc* expression.

### GENE EXPRESSION IN WILD TYPE AND *35S:MtFTa1* TRANSGENIC *Medicago* PLANTS IN LONG DAY CONDITIONS

**FIGURE 5:**
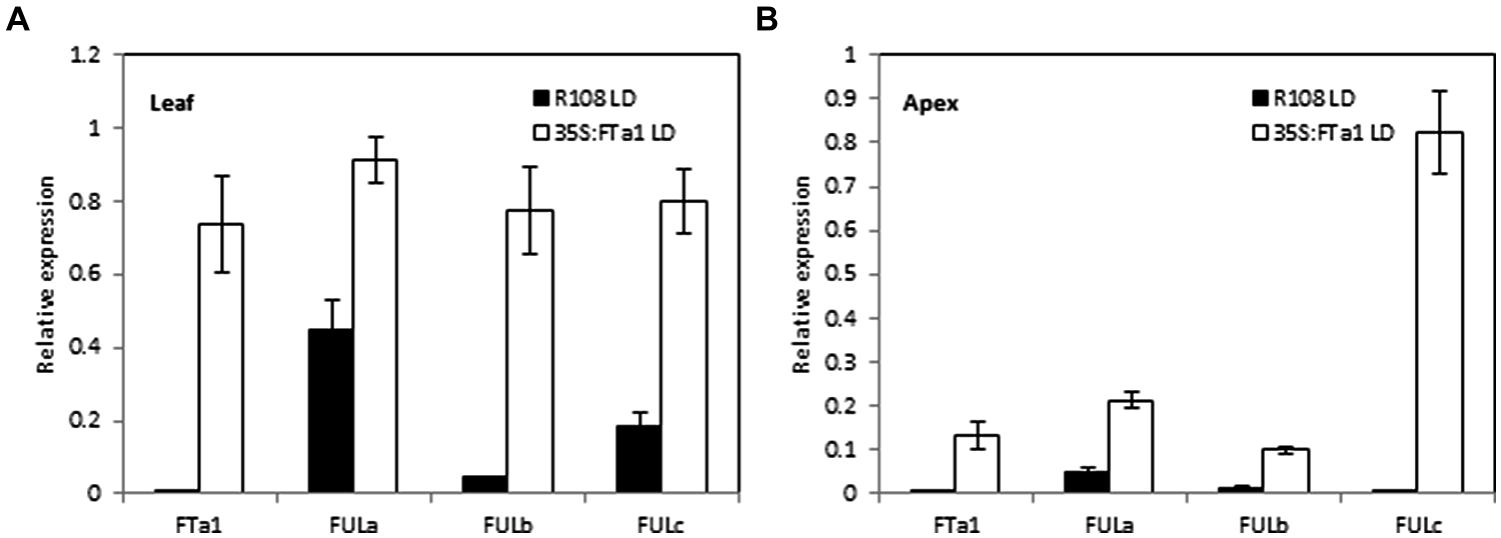
**Effect of ectopic expression of the *MtFTa1* gene on *MtFULa*, *MtFULb,* and *MtFULc* transcript levels in *35S:MtFTa1* transgenic Medicago R108 plants in LD conditions.** Relative gene expression levels in trifoliate leaves **(A)** and uppermost apical buds **(B)** in *35S:MtFTa1* and wild type R108 (14 day-old) plants grown in LD. Tissues were harvested at ZT2. Gene expression was determined using RT-qPCR and the data are shown as the mean ± SE of three biological replicates, which were normalized to *PDF2*. The data is presented relative to the highest value in both tissues for each gene.

The experiments above using the *fta1* mutant indicated that all the three *MtFUL* genes were dependent on *MtFTa1* for maximal transcript accumulation. In addition, in previous work we showed that *MtFULb* levels were elevated in LD plants over expressing *MtFTa1* (Yeoh et al., 2013). Here, we examined the effect of *35S:MtFTa1* on *MtFULa*, *MtFULb,* and *MtFULc* levels in young transgenic *Medicago* plants grown in LD conditions (**Figure 5**). No floral buds were visible on the plants. *MtFTa1* transcript was detected at very high levels in the leaves and apices of the transgenic line compared to wild type R108. The effect of *MtFTa1* overexpression on *MtFULa* levels was quite modest. *MtFULa* was quite abundant in wild type plants in LD, but its expression level increased particularly in the shoot apex of the transgenic plants. A stronger effect was observed on the *MtFULb* transcript levels in the leaf of *35S:MtFTa1* plants. The effect on *MtFULc* transcript levels was quite strong in the leaf, but even more striking was the massive increase of *MtFULc* levels in the shoot apical samples of the transgenic line compared to wild type.

### EFFECT OF *35S:MtFUL* TRANSGENES ON *Arabidopsis* FLOWERING TIME AND INFLORESCENCE DEVELOPMENT

**FIGURE 6:**
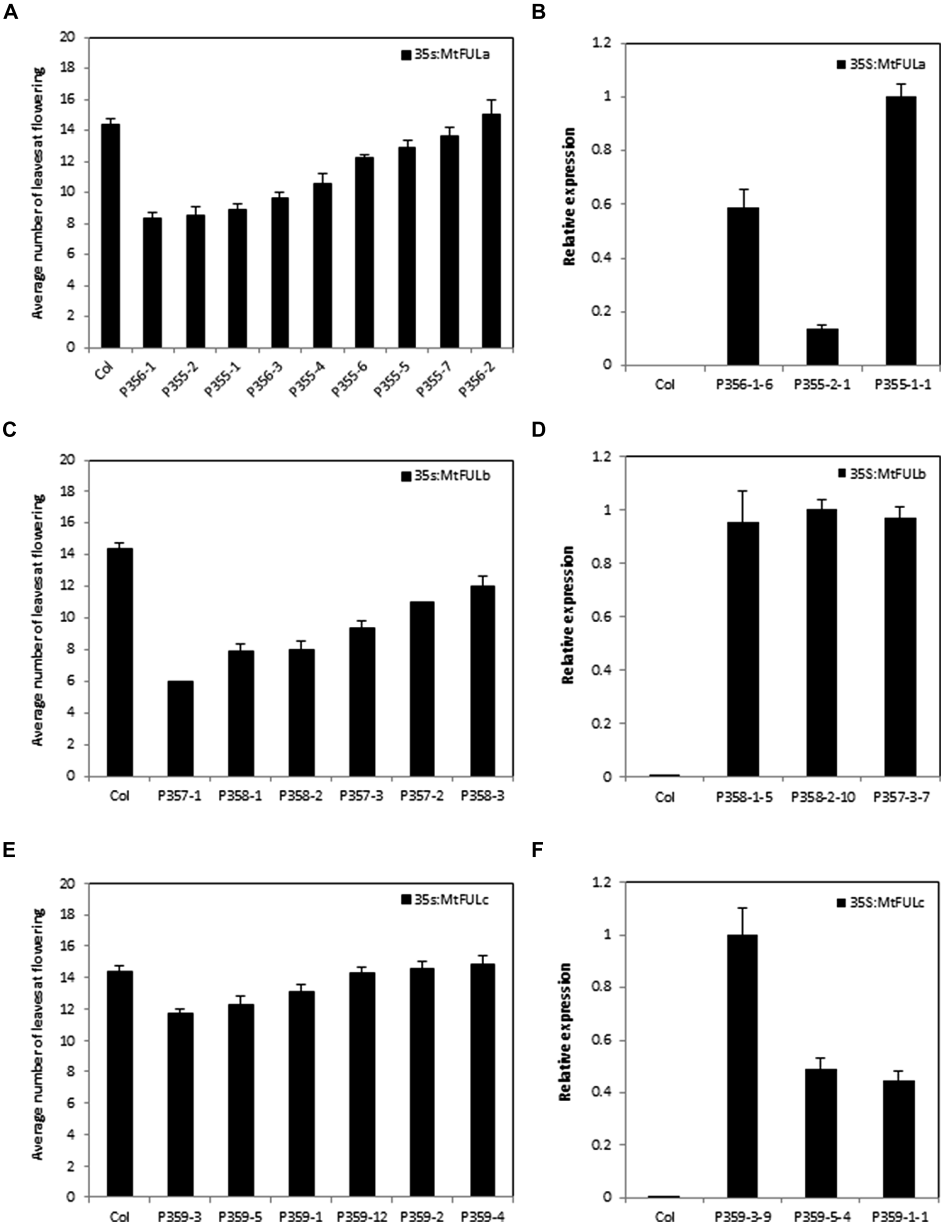
**Flowering time and gene expression in T2 transgenic *Arabidopsis* plants with *35S:MtFUL* transgenes in LD conditions.** Graphs showing the flowering time observed in T2 transgenic families of *35S:MtFULa* **(A)**, *35S:MtFULb* **(C)**, and *35S:MtFULc* **(E)** compared to wild type Columbia plants. Plants were grown in LD until they flowered. Flowering time was measured as the total number of rosette and cauline leaves at flowering. The T2 flowering time data is presented as the mean ± SE where *n* = 3–10 plants except for P357-1, 2 where *n* = 1. Gene expression of *MtFULa* **(B)**, *MtFULb* **(D),** and *MtFULc* **(F)** in transgenic *35S:MtFUL Arabidopsis* plants compared to wild type Columbia plants. Gene expression was determined in individual T2 plants from representative lines using RT-qPCR and normalized to At2g32170. The data are shown as the mean ± SE of three PCR technical replicates. The data is presented relative to the plant with the highest transgene expression for each over-expression construct.

**FIGURE 7:**
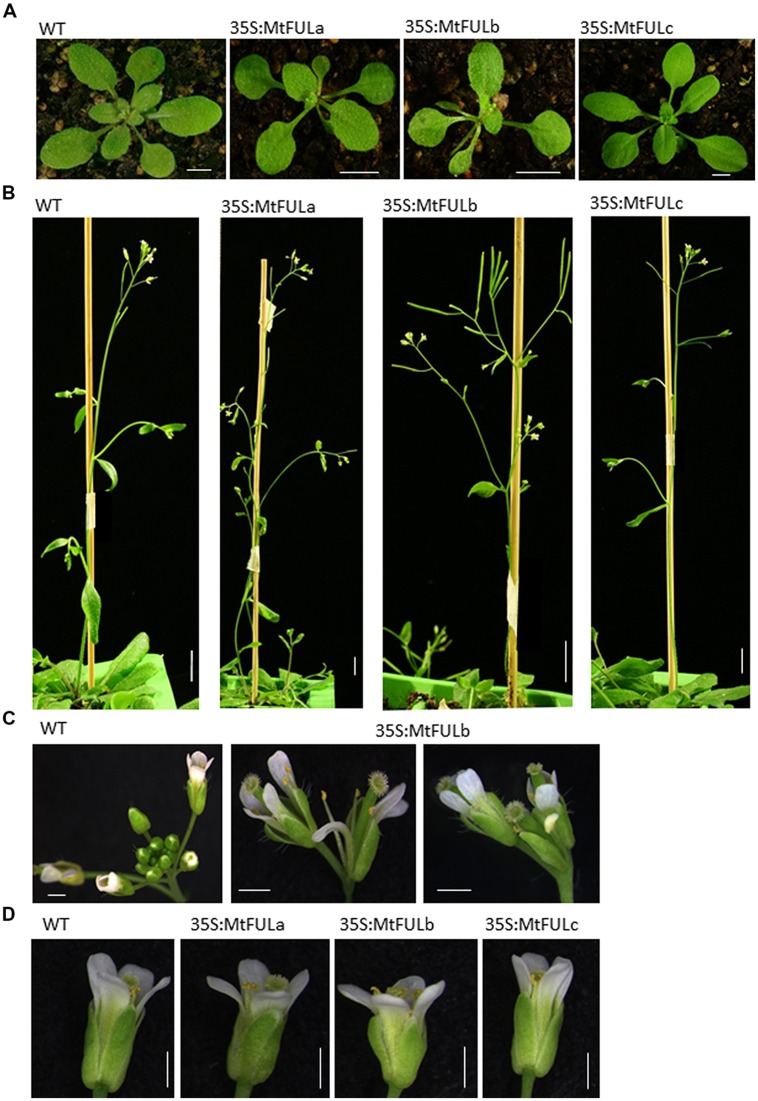
**Photographs of plant architecture, inflorescence, and floral phenotypes of T2 *Arabidopsis* plants with *35S:MtFUL* transgenes in LD conditions. (A)** *35S:MtFULa*, *35S:MtFULb,* and *35S:MtFULc* plants and wild type Col at the time of the transition to flowering. **(B)** The terminal flower phenotype observed in some *35S:MtFULb* plants compared to Col inflorescence apices. **(C)** The short stature and low number of siliques produced by some *35S:MtFULb* plants due to the premature termination of primary inflorescence development. **(D)** Small flowers produced by some of the *MtFUL* transgenics compared to wild type Col. Scale bars are **A**: 5 mm, **B**: 1 cm, **C,D**: 1 mm.

To investigate the effect of *35S:MtFUL* transgenes on *Arabidopsis* flowering, cDNA clones of *MtFULa*, *MtFULb,* and *MtFULc* genes were obtained by PCR amplification with gene specific primers. The cDNA of each of the three *MtFUL* genes was then inserted into a GATEWAY binary vector conferring resistance to the herbicide Basta and introduced into Col wild type plants. Transgenic T1 plants (>10 for each construct) were selected by spraying with Basta. T2 seeds were grown up from 6 to 9 independent transgenic lines and Basta-resistant plants were again selected, genotyped and phenotyped (**Figures 6** and **7**).

Flowering time was determined for individual plants from each T2 family and for wild type Col by counting the total number of rosette and cauline leaves (**Figure 6**). A range of flowering times were observed in the *MtFULa* and *MtFULb* families with some lines flowering earlier than wild type Col (**Figures 6A,C and 7A**). The earliest *35S:MtFULa* families flowered at ∼8 leaves on average compared to the wild type average of ∼14 leaves, while one *35S:MtFULb* plant flowered with six leaves. The *35S:MtFULc* lines flowered in a more similar manner to wild type overall, with only two lines slightly earlier than wild type (**Figure 6E**). RT-qPCR analysis on a selection of the transgenic plants confirmed that the transgenes were overexpressed in the *35S:MtFUL* transgenic *Arabidopsis* plants (**Figures 6B,D,F**).

After bolting, a striking terminal flower phenotype became apparent in some of the *35S:MtFULb* lines that was not observed in the *35S:MtFULa*, *35S:MtFULc,* or wild type Col plants (**Figures 7B,C**). Plants from independent transgenic *35S:MtFULb* families had shorter primary inflorescences than wild type because of the termination of inflorescence development and production of a terminal flower (**Figures 7B,C**). We also observed that single flowers and floral organs of some transgenic plants were smaller than wild type (**Figure 7D**). To quantify this, we measured sepal length and petal width on photographs of flowers taken from two-three independent transgenic lines of *35S:MtFULa, 35S:MtFULb,* and* 35S:MtFULc* and wild type Col. A total of 23–30 sepals and 7–10 petals were measured from each group of transgenic lines with 10 and 7 wild type sepals and petals measured, respectively. Sepals from both *35S:MtFULa* (1.84 mm ± 0.08 to 0.05 SE) and *35S:MtFULb* (1.6 mm ± 0.07 to 0.05 SE) transgenic plants were significantly shorter than wild type Col sepals (2.06 mm ± 0.12 to 0.05 SE). Sepals from *35S:MtFULc* flowers (1.97 mm ± 0.06 to 0.05 SE) were not significantly different from Col. Petals from *35S:MtFULa* (0.6 mm ± 0.06 to 0.05 SE) and *35S:MtFULb* (0.54 mm ± 0.04 to 0.05 SE) plants were also narrower than wild type Col petals (0.72 mm ± 0.08 to 0.05 SE) with *35S:MtFULb* sepals significantly different from wild type. Petals from *35S:MtFULc* (0.72 mm ± 0.08 to 0.05 SE) were similar to wild type petals. Finally, when seeds were harvested, we observed that all six of the *35S:MtFULb* lines had indehiscent (non-shattering) siliques with low numbers of small, dark-colored seeds, one of seven lines of *35S:MtFULa* had siliques that were non-shattering, while all six of the *35S:MtFULc* lines had siliques that shattered like wild type Col.

## DISCUSSION

In this study, the phylogenetic analysis we performed with the three *Medicago* FUL-like genes using predicted full-length protein sequences is consistent overall with the recent extensive phylogenetic analysis of AP1/SQUA/FUL proteins (Berbel et al., 2012). As shown in their tree, MtFULa and MtFULb form a sister clade to AtFUL, with all three proteins placed in the eudicot FUL clade (Litt and Irish, 2003). Although MtFULc identified *Arabidopsis* FUL as the top hit in our BLAST searches of the *Arabidopsis* protein database, we and Berbel et al. (2012) found that it and the orthologous pea VEG1/PsFULc claded with AGL79, a divergent *Arabidopsis* paralog of FUL (Pabón-Mora et al., 2012). Our BLAST searches of the *Medicago* protein database identified two new MADS-box proteins that clustered tightly with *Arabidopsis* AP1/MtPIM (Benlloch et al., 2006). All the three *MtFUL* genes have closely related pea homologs. The existence of multiple *Medicago* and pea *FUL* genes as opposed to one gene in *Arabidopsis*, appears to be a similar case to our recent identification of duplicated *SVP* MADS factors in *Medicago* (Jaudal et al., 2014).

Gene expression profiling indicated that the three *MtFUL* genes have potential roles in flowering time, inflorescence identity and/or flower development. In LD, transcripts of *MtFULa*, *MtFULb,* and the floral integrator *MtFTa1* increased in leaves and/or shoot apices of wild type plants prior to the transition to flowering, while *MtFULc* transcript strongly increased in shoot apices after flowering had occurred. Expression of all of the genes was detected in floral buds and open flowers. Of the three *MtFUL* genes, *MtFULb* had the most similar pattern of expression to *MtFTa1*. However, it is likely that *MtFULa* and *MtFULb* genes share partially overlapping functions given their sequence conservation, similar expression profiles and promotive effects on *Arabidopsis* flowering time. Similar results were observed when the transition to flowering was accelerated in LD by vernalization of germinated seeds. Transcripts levels of *MtFTa1* and the three *MtFUL* genes were very low in the newly germinated seeds and during the cold treatment, but expression levels began to rise prior to flowering and when the plants were grown in warm LD conditions. Thus none of *MtFUL* genes are directly regulated by cold. This is unlike the temperate grass flowering time regulator, *VRN1,* from the* FUL-like* clade in the AP1/SQUA/FUL lineage, whose transcript levels rise during vernalization (Trevaskis et al., 2007; Distelfeld et al., 2009; Berbel et al., 2012).

In *Arabidopsis*, *FUL* is most prominently expressed in the inflorescence apex at the transition to flowering and in the valves of the developing carpel (Mandel and Yanofsky, 1995a; Gu et al., 1998). Induction of flowering with LD photoperiods results in a rapid increase in *FUL* expression in the shoot meristem and in young leaf primordia, from a very low level in rosette leaves (Hempel et al., 1997; Gu et al., 1998; Ferrándiz et al., 2000a). Transcript is not present in early floral primordia, but appears from about stage 3 onward and is also detected in the vasculature of stems and cauline leaves, but not in roots (Gu et al., 1998). In comparison to *Arabidopsis FUL*, *MtFULc* has the most similar expression pattern with high expression in the shoot apex, flower buds, and flowers after the transition to flowering. *MtFULa* and *MtFULb* have rather a different profile from *Arabidopsis FUL* in some aspects as they are both expressed abundantly in leaves before the transition to flowering.

Do the *MtFUL* genes promote the transition to flowering in *Arabidopsis*? The *35S:MtFULa* and *35S:MtFULb* gene expression constructs accelerated flowering in wild type *Arabidopsis* in LD in some independent transgenic lines. The effect of *35S:MtFULa* and *35S:MtFULb* on *Arabidopsis* flowering time contrasts with our previous over expression of two *MtSVP* MADS-box genes that delayed flowering in *Arabidopsis* (Jaudal et al., 2014). Thus, these findings suggest that *MtFULa* and *MtFULb* have functional roles in controlling flowering time as displayed by *AtFUL* (Gu et al., 1998; Ferrándiz et al., 2000b; Melzer et al., 2008; Balanzà et al., 2014). This is further supported by the fact that sustained upregulation of *MtFULa* and *MtFULb* requires *MtFTa1,* a key regulator of flowering time in *Medicago* (Laurie et al., 2011). It is highly probable that *MtFULa* and *MtFULb* are downstream targets of *MtFTa1* because their expression levels were lower in the* fta1* mutant compared to wild type and both genes were upregulated in transgenic plants over expressing *MtFTa1.* As shown in *Arabidopsis*, FT directly activates the floral identity genes *FUL* and *AP1* (Teper-Bamnolker and Samach, 2005).

Aside from early flowering, a striking additional phenotype was observed in some *35S:MtFULb* transgenic *Arabidopsis* plants, which has resemblance with *35S:AtFUL* transgenics (Ferrándiz et al., 2000b). These plants produced a terminal flower, similar to a *tfl1* mutant, causing premature termination of inflorescence development. This also bears similarity to over expression of the *FUL*-related gene *Arabidopsis AP1* which results in very early flowering and terminal flowers in transgenic *Arabidopsis* due to repression of *TFL1* (Mandel and Yanofsky, 1995b; Ferrándiz et al., 2000a). Our results indicate that *35S:MtFULb* may negatively regulate *TFL1* in *Arabidopsis* causing a *tfl1* mutant phenotype. Over expression of a chrysanthemum *FUL-like* gene *CIM8* also accelerated flowering and led to production of terminal flowers in *Arabidopsis* (Wang et al., 2013). This was accompanied by increased expression of *FT, AP1*, and* LFY*, leading the authors to suggest that *CIM8* positively regulates *FT* in *Arabidopsis*. It is possible that *MtFULa* and *MtFULb* regulate *MtFTa1* in* Medicago* because the onset of their expression occurs before *MtFTa1* in our time-course experiments. We also observed that siliques of the *35S:MtFULb* transgenic plants exhibited an indehiscent (non-shattering) phenotype similar to *35S:AtFUL Arabidopsis* siliques due to repression of *SHP1/2* genes (Ferrándiz et al., 2000b; Ferrándiz and Fourquin, 2014).

In contrast to *MtFULa* and *MtFULb,* overexpression of* MtFULc* had only very mild effects in promoting flowering time of transgenic *Arabidopsis*. Thus, it is possible that *MtFULc* is not involved in regulating floral induction, but in other aspects of plant development, although the *Arabidopsis* transgenic plants resembled wild type plants in aerial architecture and development. However, this is in fact shown in pea *VEG1/PsFULc*, which is not involved in flowering time control in pea, but is expressed predominantly during and after the floral transition in the shoot apex where it has an important function in specifying secondary inflorescence meristem identity necessary for compound inflorescence development, rather than the simple raceme of *Arabidopsis* (Berbel et al., 2012). *VEG1* acts downstream of* GIGAS* (the pea *FTa1* gene) because *VEG1* was not expressed in *gigas* mutants (Berbel et al., 2012). This might also be the case for *MtFULc* because its expression is highly dependent on functional *MtFTa1* with almost no expression detected in the *fta1* mutant throughout development and overexpression of *MtFTa1* also led to upregulation of *MtFULc.*

The implications of our work overall are that the *MtFUL* genes are all regulated by *MtFTa1* and that *MtFULa* and *MtFULb* are likely to play a role in promoting the transition to flowering in *Medicago*. In addition, we observed previously that *MtFULb* transcript accumulation in young *Medicago* plants was increased in leaves by inductive LD photoperiods compared to short days, consistent with a role in flowering time control (Jaudal et al., 2013). On the other hand, *MtFULc* may have a similar role to pea *VEG1/PsMtFULc* in compound inflorescence development in *Medicago*. The overlapping and distinct *MtFUL* expression patterns in vegetative and reproductive development suggest that they have other roles in *Medicago* development. The next steps would be to determine the functional roles of the *MtFUL* genes in *Medicago*. In particular, it will be interesting to know if the *MtFUL* genes regulate *MtFTa1* and whether they interact with *MtSOC1* genes to regulate flowering time and perennial traits.

## AUTHOR CONTRIBUTIONS

JP and MJ: conceived and designed the experiments. MJ, LZ, and CC: performed the experiments. JP, MJ, LZ, and CC: analyzed the data. JP and MJ: wrote the paper.

## Conflict of Interest Statement

The authors declare that the research was conducted in the absence of any commercial or financial relationships that could be construed as a potential conflict of interest.

## Footnotes

1 http://www.jcvi.org/medicago/

2 http://www.arabidopsis.org/

3 http://www.ncbi.nlm.nih.gov/
